# Anti-Realist Pluralism: a New Approach to Folk Metaethics

**DOI:** 10.1007/s13164-019-00447-8

**Published:** 2019-09-05

**Authors:** Thomas Pölzler, Jennifer Cole Wright

**Affiliations:** 1 Department of Philosophy, University of Graz, Attemsgasse 25/II, 8010 Graz, Austria; 2 Department of Psychology, College of Charleston, 57 Coming Street, Charleston, SC 29424 USA

## Abstract

Many metaethicists agree that as ordinary people experience morality as a realm of objective truths, we have a prima facie reason to believe that it actually is such a realm. Recently, worries have been raised about the validity of the extant psychological research on this argument’s empirical hypothesis. Our aim is to advance this research, taking these worries into account. First, we propose a new experimental design for measuring folk intuitions about moral objectivity that may serve as an inspiration for future studies. Then we report and discuss the results of a survey that was based on this design. In our study, most of our participants denied the existence of objective truths about most or all moral issues. In particular, many of them had the intuition that whether moral sentences are true depends both on their own moral beliefs and on the dominant moral beliefs within their culture (“anti-realist pluralism”). This finding suggests that the realist presumptive argument may have to be rejected and that instead anti-realism may have a presumption in its favor.

## Introduction

Are there things that are morally right or wrong, good or bad, virtuous or vicious, and so on independently of what anybody thinks about them? Moral realists believe that such objective moral truths exist (e.g., Brink 1989; Huemer 2005; Shafer-Landau 2003). Anti-realists, in contrast, deny them. As they see it, moral sentences are either not truth-apt at all (non-cognitivism; Ayer 1952; Blackburn 2000); are all false (error theory; Joyce 2001; Mackie 2011); or are, if true, only subjectively true (subjectivism; Harman 1996; Prinz 2007).^1^

Several metaethical arguments appeal to what ordinary people think about the existence of objective moral truths. Most important among them is the widely held “presumptive argument” (e.g., Brink 1989; Enoch 2017a, 2017b; Dancy 1986; Huemer 2005; McNaughton 1988). According to this argument, it seems to ordinary people that morality is a realm of objective truths; therefore we have a prima facie reason to believe that it actually is such a realm.^2^ McNaughton, for example, writes:

The realist insists on an obvious, but crucial, methodological point: there is a presumption that things are the way we experience them as being […]. Moral value is presented to us as something independent of our beliefs or feelings about it; something which may require careful thought or attention to be discovered. There is a presumption, therefore, that there is a moral reality to which we can be genuinely sensitive. (McNaughton 1988: 40)

Despite the presumptive argument’s importance, philosophers have so far not provided any solid empirical evidence for or against its underlying hypothesis that ordinary people experience morality as a realm of objective truths. At first sight recent research in psychology promises to remedy for this shortcoming.

In the last 15 years an increasing number of psychologists have begun to study folk intuitions about the existence of objective moral truths. Their results suggest that rather than being realists, ordinary people intuitively tend towards “metaethical pluralism” (Wright et al. 2013; Wright 2018): they regard moral realism as true with regard to some moral sentences or circumstances and anti-realism as true with regard to other moral sentences or circumstances (e.g., Beebe 2014; Beebe and Sackris 2016; Sarkissian et al. 2011; Wright et al. 2013, 2014; Wright 2018). In light of this finding it is tempting to regard the presumptive argument as empirically implausible (and likewise any other argument that presupposes dominantly realist intuitions). However, such a conclusion may be premature.

A number of serious doubts have been raised about the validity of the existing psychological studies on folk moral realism. Most importantly, it has been argued that many of these studies failed to fully and exclusively measure what they set out to measure. They have not adequately methodologically distinguished between moral realism and anti-realism; have conflated these positions with distinct metaethical positions; have elicited irrelevant intuitions (such as first-order moral intuitions); and so on (see Beebe 2015; Pölzler 2018a, 2018b). This means that there are important improvements to be made or follow-up studies to be conducted before psychological findings can be appealed to as evidence for or against philosophers’ hypotheses about folk moral objectivity.

The aim of this paper is to advance psychological research on folk moral realism, and in a way that is likely to allow for philosophical conclusions. We begin by introducing previous research and explain why it has lacked in validity (Section 1). Then we propose a new experimental approach designed to overcome these worries as far as methodologically feasible (Section 2). Finally, and most importantly, we report and discuss the results of a psychological study that was based on this new design. In this study, most of our participants neither favored realism nor pluralism, but rather denied the existence of objective moral truths *tout court*. In particular, many of them had the intuition that whether moral sentences are true depends both on their own moral beliefs and on the dominant moral beliefs within their culture (“anti-realist pluralism”) (Sections 3 and 4). This suggests that realists’ presumptive argument may indeed have to be rejected and that instead anti-realism may have a presumption in its favor.

## Previous Research

In the last 15 years psychologists have conducted a number of studies on folk moral objectivity, typically labelled as being about realism (this is the label we will use), objectivism, subjectivism or relativism (e.g., Beebe 2014, 2015; Beebe and Sackris 2016; Beebe et al. 2015; Cova and Ravat 2008; Fisher et al. forthcoming; Goodwin and Darley 2008, 2010, 2012; Heiphetz and Young forthcoming; Nichols 2004; Nichols and Folds-Bennett 2003; Sarkissian et al. 2011; Wainryb et al. 2004; Wright 2018; Wright et al. 2013, 2014; Young and Durwin 2013). In this section we introduce the methods of these studies and then recapitulate several worries about them.

### Methods

The terms “moral truth” and “moral objectivity” can be understood in different ways (see, e.g., Lynch 2013; Raz 2001). In order for psychological research on folk moral realism to possibly bear on metaethics it must assume roughly the same understanding as this area of philosophical inquiry. So what do metaethicists mean when they discuss moral truth and moral objectivity? Here we assume a correspondence theory of moral truth and a mind-independence conception of moral objectivity. That is, we take the moral realism/anti-realism debate to be about whether moral sentences are true in that they match (objective) moral facts; and about whether these facts are objective in that they would obtain even if observers had different or no mental states towards them (see, e.g., Huemer 2005; Joyce 2007).

So far psychologists have mainly attempted to measure realist and anti-realist intuitions by what we will henceforth refer to as “disagreement tasks” and “truth-aptness tasks”. Disagreement tasks have been the most common measure. In their study on children between the age of five and nine Wainryb et al. (2004), for example, asked participants to imagine two persons who hold different moral beliefs. Then participants were asked to interpret this scenario.

Sarah believes that it’s okay to hit and kick other children, and Sophie believes that it’s wrong to hit and kick other children. […] Do you think that only one belief [what Sophie believes] is right, or do you think that both beliefs [what both Sophie and Sarah believe] are right? (Wainryb et al. 2004: 691)

According to Wainryb et al., “only one belief is right” responses express realist intuitions, and “both beliefs are right” responses express anti-realist intuitions (2004: 692).^3^

Truth-aptness tasks have been introduced by Goodwin and Darley. In their influential 2008 study participants were presented a moral sentence (e.g., “Anonymously donating a significant proportion of one’s income to charity is a morally good action”; 2008: 1361–1362). Then they were asked, among others, whether they take this sentence to be “true”, “false” or “an opinion or attitude“:

How would you regard the previous statement? Circle the number. (1) True statement. (2) False statement. (3) An opinion or attitude. (Goodwin and Darley 2008: 1344)

In Goodwin and Darley’s interpretation, both “true” and “false” responses express realist intuitions, and “opinion or attitude” responses express intuitions in favor of subjectivism (see 2008: 1345).^4^

### Worries

One minor problem with research such as Wainryb et al.’s and Goodwin and Darley’s is that it has lacked in external validity. Many studies have mainly surveyed US students (e.g., Goodwin and Darley 2008; Wright et al. 2013),^5^ have mainly used particular kinds of item statements (e.g., such as statements that involve “thin” moral concepts and action types) (e.g., Beebe and Sackris 2016; Goodwin and Darley 2008; Nichols 2004),^6^ or have involved unrealistic or humorous experimental stimuli (e.g., Sarkissian et al. 2011).

In addition, this research is also subject to a more fundamental worry about its construct validity. Researchers have typically assumed adequate definitions of moral realism and anti-realism; that is, they have understood these positions as affirming or denying the existence of objective moral truths in the sense explained above (e.g., Goodwin and Darley 2008; Nichols 2004). But these definitions have then not been properly operationalized. This means that their research likely failed to fully and exclusively measure participants’ intuitions about the existence of objective moral truths. It may not provide helpful information about rough or coarse-grained or pre-reflective intuitions of this kind.

Elsewhere one of us has identified ten common sources of this (potential) lack in construct validity (Pölzler 2018a, 2018b). Many of them straightforwardly apply to the measures by Wainryb et al. and Goodwin and Darley introduced above.^7^

1. *Failure to Cover Variants of Anti-Realism*
  Most studies’ answer options have failed to cover main variants of anti-realism (i.e., subjectivism, error theory or non-cognitivism). Participants who felt drawn towards these variants hence had to respond in ways that did not reflect their actual intuitions — which may have been ways that were classified as realist.
2. *Failure to Cover Variants of Subjectivism*
  Many studies also have not sufficiently considered variants of subjectivism that may plausibly be held by ordinary people (i.e., cultural relativism and individual subjectivism). Again, this may have systematically distorted their results.
3. *Conflation with Cognitivism/Non-Cognitivism Debate*
  With regard to truth-aptness tasks, researchers have assumed that by measuring intuitions about the truth-aptness of moral sentences (cognitivism/non-cognitivism debate) they can illuminate intuitions about moral realism in a comprehensive sense. But anti-realism is compatible with both moral sentences being truth-apt and not being truth-apt.
4. *Conflation with Universalism/Relativism Debate*
  The realism/anti-realism debate has occasionally also been conflated with the debate about the scope of moral sentences; that is, about how widely these sentences apply (universalism/relativism debate).
5. *Elicitation of Irrelevant First-Order Moral Intuitions*
  Even though moral realism and anti-realism are claims in metaethics, and hence descriptive, some measures have prompted first-order moral intuitions. These intuitions may partly explain participants’ answers.
6. *Elicitation of Irrelevant Epistemic Intuitions*
  Participants’ responses may also have been unduly systematically influenced by epistemic intuitions; that is, intuitions about whether persons know, are justified in believing or are certain about moral propositions.
7. *Ex ante Classification of Statements*
  So far most studies’ item statements have been classified as moral or non-moral by the researchers themselves. Participants’ responses therefore may not only be explained by their realist or anti-realist intuitions but also by their not having regarded some statement as moral at all.
8. *Misleading or Biasing Instructions*
  Some studies have involved misleading or biasing instructions of the realism/anti-realism debate or their truth-aptness or disagreement tasks.
9. *Unsubstantiated Assumption about Moral Truth*
  So far no evidence has been provided for the claim that participants understand studies’ underlying concepts of truth, rightness or correctness in a correspondence theoretic sense.
10. *Unsubstantiated Identification of Responses with Intuitions*
  It is also possible that participants have systematically conflated the studies’ questions with independent questions (such as about the amount of societal consensus about moral sentences) or that they do not have any determinate intuitions about the existence of objective moral truths at all.

While some of the existing research (e.g., Sarkissian et al. 2011; Wright 2018) has managed to escape some of the above worries, none of the studies conducted thus far emerge completely unscathed. In light of these worries it seems reasonable to conclude that the extant psychological research, while uncovering many interesting aspects of people’s underlying moral psychology, has thus far failed to yield data that can conclusively weigh in as evidence about the content of folk metaethical intuitions. More careful work needs to be done before intuitions can be classified according to metaethical categories and philosophical conclusions can possibly be drawn.

## New Experimental Design

To improve upon the situation described in the previous section, we developed a new experimental design for measuring folk intuitions about moral objectivity. This design purports to overcome W1 to W10 as far as methodologically feasible.^8^ Even though it is not perfect it constitutes an important first step towards greater construct validity and may serve as an inspiration for future research. Our new experimental design involves three main parts: (1) instructions, (2) abstract tasks, and (3) concrete tasks. In what follows we will explain each of these parts and then provide some general discussion.

### Instructions

Our design starts with an explanation that purports to prevent participants from misreading its main tasks as asking for first-order moral intuitions (W5) or other matters that are unrelated to the issue of moral objectivity (W10). To begin with, we explain the difference between what we call “normative” and “meta-ethical” sentences about morality:

Normative sentences about morality express moral judgments. In uttering these sentences we evaluate something morally; we indicate that we regard something as morally right or wrong, good or bad, virtuous or vicious, and so on.

[…]

Meta-ethical sentences about morality do not express moral judgments. In uttering them we remain evaluatively neutral. Instead, we are making claims about the nature of morality itself.

This explanation is supplemented by the least controversial and least biasing examples of normative and metaethical sentences that we could think of (W8).^9^ We also explicitly note that the classification of sentences as normative or metaethical is independent from the extent to which one agrees to them. Finally, participants are informed that the present study concerns *metaethical* sentences and that they are therefore asked to abstract from their first-order moral intuitions.

Given that we are interested in your intuitions about meta-ethical sentences, we ask you to “bracket” your views about the normative sentences that we will present you (to ignore these intuitions or put them to the side). For the purposes of this study it does not matter whether, for example, you judge that breaking promises is wrong, that the US has a duty to reduce their greenhouse gas emissions, and so on.

Following these explanations, we test and improve participants’ understanding of the normative/metaethical distinction by two comprehension checks: (1) a theoretical question about what they have just read (right answer: “Normative sentences express moral judgments and meta-ethical sentences make claims about the nature of morality itself”), and (2) an exercise that asks them to classify several sentences as either normative or metaethical (e.g., “Eating meat is morally wrong” and “Gaining moral knowledge requires careful rational reflection”). Participants who do not fully succeed on either check are presented the above explanations once again and have to complete the relevant check/s one more time.

### Abstract Measures

Previous research on folk moral realism has exclusively measured responses to concrete moral sentences.^10^ This experimental design, in contrast, also involves abstract tasks (formulated as being about morality in general). Our motivation for including such tasks is threefold. First, abstract responses are less likely to be influenced by first-order moral intuitions. Second, folk intuitions about moral responsibility, knowledge and other non-metaethical philosophical issues have been found to vary across the abstract/concrete distinction (e.g., Nichols and Knobe 2008; Sinnott-Armstrong 2008). And third, no single measure of folk metaethical intuitions will ever be perfect. So the more comprehensive and varied one’s measures, the more robust and complete (and thus valid) the picture one can gain from them.

The five abstract tasks that we developed are (1) a theory task, (2) a metaphor task, (3) a comparison task, (4) a truth-aptness task, and (5) a disagreement task.

#### Theory Task

The theory task begins with an explanation of the three main questions that determine one’s position in the moral realism/anti-realism debate: Do moral sentences intend to state moral facts? If yes, do these facts exist? And if yes, are they independent from what anybody thinks about them? Then participants are asked to choose their preferred answer to these questions, with their options corresponding to several main variants of moral realism and anti-realism (W1), including what we take to be the most plausibly held variants of subjectivism (cultural relativism and individual subjectivism) (W2).

- When a person says that something is morally right or wrong, good or bad, etc. she intends to state a fact. Such facts exist – and they are independent from what anybody thinks about them. For example, an action that is morally wrong is wrong no matter what anyone thinks. So it would still be wrong even if you yourself, or the majority of the members of your culture, thought that it is not morally wrong. [SECULAR REALISM]
- When a person says that something is morally right or wrong, good or bad, etc. she intends to state a fact. Such facts exist – and they depend on God’s will. For example, an action is only morally wrong if God forbids us to perform the action. If God did not forbid us performing the action, it would not be wrong. [THEIST REALISM]
- When a person says that something is morally right or wrong, good or bad, etc. she intends to state a fact. Such facts exist – and they depend on what the majority of the members of her culture think about them. For example, an action is only morally wrong if the majority of the members of your culture believe that it is wrong. If the majority of the members of her culture did not believe the action to be wrong, it would not be wrong. [CULTURAL RELATIVISM]
- When a person says that something is morally right or wrong, good or bad, etc. she intends to state a fact. Such facts exist – and they depend on what individuals think about them. For example, an action is only morally wrong if you yourself believe that it is morally wrong. If you did not believe the action to be wrong, it would not be wrong. [INDIVIDUAL SUBJECTIVISM]
- When a person says that something is morally right or wrong, good or bad, etc. she intends to state a fact. Such facts do not exist. Thus, it is never the case that something is morally right or wrong, good or bad, etc. No such moral statement can be true. [ERROR THEORY]
- When a person says that something is morally right or wrong, good or bad, etc. she does not intend to state a fact. Instead, she intends to communicate/express her feelings, emotions, intentions or attitudes about it. For example, by saying that some-thing is wrong, you only express feelings of disapproval towards it (and that is the only thing you are doing). Moreover, there are no facts about what is morally right or wrong, good or bad, etc. [NON-COGNITIVISM]

#### Metaphor Task

Moral realism and anti-realism are claims about moral facts. Our second abstract task asks participants to choose between a number of metaphors for these facts which correspond to the main variants of realism and anti-realism (W1, W2).^11^

This task is about moral facts. Moral facts are facts about what is morally right or wrong, good or bad, virtuous or vicious, and so on. For example, it could be a moral fact that it is (or is not) wrong to break promises, or that the US has (or does not have) a duty to reduce their greenhouse gas emissions. Below moral facts are explained in terms of several metaphors. Which of these metaphors seems most appropriate to you?

- Moral facts are “discovered”. They can be discovered in the same way in which we discover other facts about the objective world. [SECULAR REALISM]
- Moral facts are “divine commandments”. They are introduced and determined by God. [THEIST REALISM]
- Moral facts are “cultural inventions”. They are introduced and determined by cultures. [CULTURAL RELATIVISM]
- Moral facts are “individual inventions”. They are introduced and determined by individuals. [INDIVIDUAL SUBJECTIVISM]
- Moral facts are “illusions”. While it may seem to us that they exist they actually do not exist at all. [ERROR THEORY or NON-COGNTIVISM]

#### Comparison Task

Next, participants are asked to which non-moral domain morality is most similar. The answer options are again formulated in such a way that they reflect the main variants of moral realism and anti-realism (W1, W2).

Below morality is being compared to various types of matters. Please indicate which comparison seems most appropriate to you.

- Morality is akin to science or mathematics. There are objective facts about what is right or wrong (facts that are independent from what anybody thinks about them). We cannot change these facts, we have to discover them. [SECULAR REALISM]
- Morality is akin to religion. What is morally right or wrong is determined by what God wants us to do. Individuals cannot, by themselves, change the moral facts. [THEIST REALISM]
- Morality is akin to social conventions. In each culture different things can be morally right or wrong. Cultures determine the moral facts. Individuals within cultures cannot, by themselves, change those facts. [CULTURAL RELATIVISM]
- Morality is akin to personal taste or preferences. For each person different things can be morally right or wrong. The individual determines the moral facts. [INDIVIDUAL SUBJECTIVISM]
- Morality is akin to superstition. It is based on a fundamental error. It assumes things exist (namely facts about rightness and wrongness) that do not actually exist. [ERROR THEORY]
- Morality is akin to exclamations (such as “Yeah!” or “That sucks!”). We use terms such as “right” and “wrong” to express our feelings, emotions, intentions or attitudes, but that is all. There are no moral facts. [NON-COGNITIVISM]

#### Disagreement Task

Our fourth task is an improved version of traditional disagreement tasks. It presents participants a case of moral disagreement about an unspecified situation and then asks them to interpret this case in ways that entail variants of moral realism and anti-realism (W1).

Consider the following scenario. Two people from the same culture are evaluating the exact same situation and utter conflicting moral sentences about it. One person says that what happened is morally bad (wrong, vicious, etc.).^12^ The other person says that what happened is not morally bad (wrong, vicious, etc.). Which interpretation of this disagreement seems most appropriate to you?

- One of these two people is right and the other one is wrong (Please note that this could be the case for several reasons: for example, because the truth of moral sentences is objective, or because it is determined by the dominant moral beliefs in their culture, or because it is determined by the commandments of God). [SECULAR REALISM, CULTURAL RELATIVISM or THEIST REALISM]
- Both people are right (because the truth of moral sentences is determined by the moral beliefs of individuals). [INDIVIDUAL SUBJECTIVISM]
- Both people are wrong (because although moral sentences intend to state moral truths, there are no such truths). [ERROR THEORY]
- Neither person is right or wrong (because moral sentences do not intend to state moral truths, and are therefore neither true nor false). [NON-COGNITIVISM]^13^

Participants who respond that one of the persons is right and the other one is wrong are presented one or two otherwise identical disagreement scenarios to discern whether they favor secular realism, cultural relativism or theist realism (W2). First they are presented a follow-up scenario in which the disagreeing parties are members of different cultures, with each of these parties’ moral judgements conforming to the dominant view within their culture. Participants who respond that under these circumstances both people are right are classified as cultural relativists. Those who again choose “One of the persons is right and the other one is wrong” are presented a third scenario in which the disagreeing parties are subject to different commands by God, with each of their moral judgements corresponding to these commands. Regarding this scenario, “One of the persons is right and the other one is wrong” answer are classified as realist and “Both people are right” answers as indicating theist realism.

#### Truth-Aptness Task

Finally, our abstract part also involves an improved version of traditional truth-aptness tasks. Participants receive an explanation of what it is for a moral sentence to be or not be truth-apt.^14^ For example, they are told that “[t]ruth-apt sentences express beliefs about facts”, and that even when we do not know whether a moral sentence is true or false it can still be truth-apt. Their understanding of this explanation is tested and improved by comprehension checks analogous to those reported in Sec. 2.1. Then participants are presented the following task (W3):

Think about moral sentences (sentences that express that something is morally good or bad, right or wrong, virtuous or vicious, and so on). Are these sentences truth-apt or not truth-apt?

- Yes, moral sentences are “truth-apt” -- that is, they intend to express how things are; what is the case (either with regard to the objective world or with regard to what particular individuals, cultures, etc. think about morality). Thus, these sentences are either true or false. [COGNITIVISM: SECULAR REALISM, THEIST REALISM, CULTURAL RELATIVISM, INDIVIDUAL SUBJECTIVISM or ERROR THEORY]
- No, moral sentences are not “truth-apt” -- that is, they do not intend to express beliefs about objective or subjective facts, but rather only express feelings, emotions, intentions or attitudes. Thus, these sentences are neither true nor false. [NON-COGNITIVISM]

### Concrete Measures

The third main part of our experimental design consists of concrete tasks. These tasks are essential to measuring people’s intuitions about moral realism because they allow conclusions about the extent to which their metaethical intuitions vary with moral sentences or circumstances. One problem with some of the previous concrete tasks has been that participants were presented purportedly moral sentences that they themselves might not have regarded as moral (W7). We therefore explain the distinction between sentences that make dominantly moral versus sentences that make dominantly non-moral evaluations (in most general and non-biasing terms).^15^ Then we ask participants to rate the following sentences as belonging to either of these categories.

- A country with the death penalty is morally worse than a country without.
- Before the third month of pregnancy, abortion for any reason is morally impermissible.
- Men who violently physically punish their children are cruel.
- John cheating (committing adultery) on his wife Elizabeth for no other reason than boredom with his marriage is morally permissible.
- Consciously discriminating against someone, by not hiring them for a job they are clearly qualified for, just because of their race is not wrong.
- It was horrendous of Martin Shkreli to overprize drugs that he knew sick people really needed.
- A world in which wealth is distributed equally is more just than a world in which it is distributed unequally.
- Martin Luther King was a righteous man.
- It is good to do onto others as you would have them do onto you.
- It is morally reprehensible of the current US administration to limit or prohibit immigration.

In the subsequent two concrete tasks participants are only presented those sentences that they themselves rated as dominantly moral. Note also that our above selection is less homogenous than that of most previous research on folk moral realism. For example, it involves sentences about particular moral cases and moral principles, sentences that involve thick moral concepts, a negated sentence, highly counterintuitive sentences, and so on. This increases the generalizability of our findings to morality as a whole.

#### Disagreement Task

In the disagreement task’s concrete version participants are asked to interpret cases of disagreement about each of their self-classified moral sentences. Here is one example:

##### Men who violently physically punish their children are cruel.

Consider the following situation. Two people from the same culture discuss whether men who violently physically punish their children are cruel. One person says that men who violently physically punish their children are cruel. The other person says that it is not the case that men who violently physically punish their children are cruel. Which interpretation of this disagreement seems most appropriate to you?

- One of these two people is right and the other one is wrong (Please note that this could be the case for several reasons: for example, because the truth of the people’s sentences is objective, or because it is determined by the dominant moral beliefs in their culture, or because it is determined by the commandments of God). [SECULAR REALISM, CULTURAL RELATIVISM or THEIST REALISM]
- Both people are right (because the truth of their sentences is determined by the moral beliefs of individuals). [INDIVIDUAL SUBJECTIVISM]
- Both people are wrong (because although their sentences intend to state moral truths, there are no such truths). [ERROR THEORY]
- Neither person is right or wrong (because their sentences do not intend to state moral truths, and are therefore neither true nor false). [NON-COGNTIVISM]

Analogously to the abstract disagreement task, answering in the first way triggers one or two follow-up scenarios (cross-cultural and God-related) that allow to distinguish secular realist from cultural relativist and theist realist intuitions.

#### Truth-Aptness Task

In the concrete truth-aptness task participants are asked to classify all of the sentences that they rated as moral as either truth-apt or not-truth-apt (W3):

Consider the following sentences. Here we are not interested in whether you believe these sentences are actually true. Our focus is rather on their truth-aptness.

Are these sentences truth-apt (i.e., they intend to express what is the case about individuals' moral beliefs, culturally dominant moral beliefs or the objective world; and thus, they are either true or false) or not truth-apt (i.e., they intend only to express feelings, emotions, intentions or attitudes, and thus, they are neither true, nor false)?

- truth-apt [COGNITIVISM: SECULAR REALISM, THEIST REALISM, CULTURAL RELATIVISM, INDIVIDUAL SUBJECTIVISM or ERROR THEORY]
- not truth-apt [NON-COGNITIVISM]

### Discussion

In its above form our new experimental design alleviates most of the worries that have been raised about previous research on folk moral realism (W1-W8). The two most fundamental worries have not yet been (fully) addressed, however; namely that participants may not understand studies’ underlying concepts of truth and rightness in a correspondence theoretic sense (W9), and that they systematically conflate their questions with independent questions or do not have any determinate intuitions about the existence of objective moral truths at all (W10).

In a pilot study we attempted to investigate whether moral truth is regarded as correspondence with facts, as coherence, or as the reaffirmation of first-order moral commitments. Participants’ verbal explanations indicated serious misunderstandings about what we were asking. The issue seemed too abstract and too intricately related to the moral realism/anti-realism debate to be addressed *en passant*. In our design we therefore only prime participants to think in correspondence-theoretic ways. For example, with regard to our truth-aptness measures, we explain that to say of moral sentences that they are truth-apt is to say that “they intend to express how things are; what is the case” (see Sec. 2.2.5 and 2.3.2).

According to the second fundamental worry, participants may systematically conflate the issue of moral objectivity with independent issues or may not have any determinate intuitions about it at all. One way in which our design addresses this worry is by involving detailed instructions of the normative ethics/metaethics and truth-aptness/not truth-aptness distinctions, and by testing participants’ understandings of these distinctions (see Sec. 2.1 and 2.2.5). In addition, we also introduced the following measures: open-ended questions that ask participants to explain some of their responses and response patterns (in particular, variation between the abstract and concrete disagreement and truth-aptness responses, and within the concrete disagreement and truth-aptness responses), completion times recordings for each task and the survey as a whole, additional comprehension checks, and attention checks.

## Study

By applying the experimental design introduced in Sec. 2 we gathered (what we deem to be) more valid and philosophically relevant data about the folk’s metaethical intuitions than previous research. In what follows we will describe the participants, methods and results of our study.

### Participants

We surveyed 172 participants. Of that, 98 participants were from Amazon MTurk (49% male) and were paid $7.25; and 74 participants were from the College of Charleston (26% male) and received two research credits.

Prior to analysis the quality of participants’ responses was assessed according to the following criteria (in the order of their weighting): (1) performance in attention checks (failure in any check automatically eliminated participants from the study), (2) study completion time, both overall and for each individual task, (3) performance in comprehension checks, (4) minimal relevance of verbal explanations. On the basis of these criteria 55 participants (32%) were excluded from analysis. For example, we did not consider the responses of participants who finished the complete survey in less than 20 min (the average time was 45 min); who copied their verbal explanations from Wikipedia and other online sources; and who took the first option in all comprehension checks.

In the end we had 117 participants, 67 from MTurk and 50 from the College of Charleston. Of these participants 63% were female; their age varied between 18 to 64 years (*M* = 29.6); and they were 86% Caucasian, 4% African American, 4% Asian American, 3% Hispanic, and 3% other.

### Methods

The survey was administered online through Qualtrics. Each participant was presented both the abstract measures (theory, metaphor, comparison, disagreement and truth-aptness tasks) and the concrete measures (disagreement and truth-aptness tasks). The order of these measures was counterbalanced, and many of the individual tasks, as well as most of the answer options, were randomly ordered. Responses to the tasks were coded as being indicative of realist or anti-realist intuitions according to the information added in square brackets in Sec. 2.

One potential worry about our methodology is that it still fails to cover many main variants of subjectivist anti-realism (W2) as well as main variants of realism (a worry that has not been addressed in Sec. 1.2 at all). This observation is correct. For example, our answer options neither reflect response-dependence theory (a main variant of subjectivist anti-realism) nor do they disentangle naturalism from non-naturalism (two main variants of secular realism). That said, it would seem methodologically overdemanding to require of any single study to cover all or even only most positions in the realism/anti-realism debate. Our study provides first helpful data because of the particular selection that we made. The positions that were included are either such that we would pre-experimentally expect them to be popular among the folk (other than, e.g., response-dependence theory) or that they necessitate additional answer options or scenarios in our tasks (other than, e.g., naturalism and non-naturalism, which are both covered by our realist answer options).^16^

Critics may also question our classification of divine command theory as a (theistic) variant of realism. According to divine command theory, a thing is morally right, good, virtuous, etc. if and only if it is commanded by God, and morally wrong, bad, vicious, etc. if and only if it is forbidden by God.^17^ This renders moral facts mind-dependent — dependent on the mind of God (e.g., Huemer 2005: 54–55). Yet, it bears noting that God may have several metaethically relevant features that most or all non-divine observers lack, such as that he/she is omni-perfect, that his/her commands never change or that these commands apply to all people at all times and places. The broad majority of metaethicists (e.g., Austin 2006; Evans 2013; Joyce 2007) hence (implicitly) define moral realism as only requiring independence from the mental states of *non-divine* observers. On this definition — which is assumed in this paper as well — divine command theory does turn out to be a variant of realism.^18^

Another important methodological question is which proportion of a participant’s intuitions about concrete cases must favor realism or anti-realism in order for the participant to count as being drawn towards these views. Elsewhere one of us has argued that from a metaethical perspective there must not be any genuine intrapersonal variation at all (Pölzler 2017, 2018b). That said, when a participant is asked to give many responses, as in our concrete tasks, the likelihood of performance errors increases. We thus stipulated that participants have “consistently” realist or anti-realist intuitions if 100% of their responses to the abstract tasks and at least 90% of their responses to the concrete tasks were in favor of only one of these views. This is the threshold above which the likelihood of realist or anti-realist intuitions having an effect on the participant’s responses is significantly greater than the likelihood of there being no such effect, as calculated by a binomial test, based on the number of separate tasks involved in each set of measures.

Finally, most of our tasks involve four anti-realist but only two realist answer options. It may be objected that this unequal distribution biases our results in favor of anti-realism. We agree that this feature is potentially problematic, especially to the extent that participants answer without sufficient effort or comprehension. But it is also hard to avoid. Our study’s most important tasks are the abstract and concrete disagreement tasks. This is because these tasks have been most widely used, have been most thoroughly refined, and are most likely to reveal more implicit intuitions, as they may be central to metaethical arguments (see Sec. 4). Prior research has shown that in order for these disagreement tasks to be minimally valid researchers need to fully tease apart metaethical positions that entail additional answer options (see W1 and W2; see also Pölzler 2018a, 2018b and fn. 17). By doing so, however, one automatically ends up with at most two options that entail realism, and a larger number of options that entail anti-realism.

To alleviate the problem of our unequally distributed answer options, we decreased insufficient effort and insufficient comprehension responding in various ways, including requiring verbal explanations from participants (see Sec. 3.1). This makes it more likely that those who opted for anti-realist options really felt drawn towards these options. Moreover, in an independent study we also confirmed that our disagreement tasks deliver plausible results for non-moral domains (scientific statements were dominantly rated as realist, and statements about social conventions and personal preferences were dominantly rated as anti-realist).^19^

### Results

#### Comprehension Checks

Our metaethics/normative ethics comprehension check involved a theoretical question and a classification exercise (see Sec. 2.1). 114 participants (97%) answered the theoretical question correctly on the first pass, and all three who got it wrong successfully corrected their answer on the second pass. The classification exercise showed a marked difference in performance between the Mturk and student participants. On average, 69% of the Mturkers classified all 14 sentences correctly at first pass, with the correction rate being 79%. In contrast, only 35% of the student participants initially classified all sentences correctly, with the correction rate being 72%.

Participants had to complete several additional comprehension and memory checks as well. 81%, 82%, 94%, and 71% correctly selected what the abstract theory, metaphor, comparison, and disagreement tasks were about; and our question of what had been presented in the concrete disagreement tasks received 83%, 75%, 75%, 72% and 77% correct responses. The MTurk participants again did somewhat better on most of these checks. This also holds for the extensive comprehension checks prior to each participant’s first truth-aptness task. While both the MTurkers and students mostly correctly answered our theoretical question (69% and 70%), the MTurkers did better at our classification exercise (48% versus 20% correct classifications at first attempt) and were able to correct more of their misclassifications (86% versus 69%).

In total, the analyzed participants’ performance in the comprehension checks was good; and where possible, mistakes were mostly corrected (indicating that participants learned from their mistakes).

#### Realist/Anti-Realist Responses

Collapsing across all of the opportunities participants had to give either a realist or an anti-realist response, and then averaging these together, we found that participants dominantly gave anti-realist responses. In response to the abstract measures the proportion of anti-realist responses was 77%, and in response to the concrete measures 89% (as opposed to 23% and 11% of realist responses) (see Tables 1 and 2). The order of the measures’ presentation did not have any significant effect; that is, participants who received the abstract measures first responded in the same way as participants who received the concrete measures first.

**Table 1:** Proportion of realist and anti-realist responses in abstract measures

	Theory	Comparison	Metaphor	Disagreement	Truth-Aptness	TOTAL (not incl. Truth-Aptness)
Realist	32%	21%	24%	14%	≤73%	23%
Anti-Realist	68%	79%	76%	86%	≥27%	77%

**Table 2:** Proportion of realist and anti-realist responses in concrete measures. Upper value = disagreement task, lower value = truth-aptness task

	Martin Luther King	Wealth Distribution	Cheating	Overpricing Drugs	Physical Punishment	Immigration	Abortion	Racial Discrimination	Death Penalty	Golden Rule	TOTAL (not incl. Truth-Aptness)
Realist	9% ≤65%	12% ≤85%	0% ≤78%	25% ≤63%	28% ≤75%	1% ≤80%	14% ≤84%	2% ≤82%	9% ≤84%	13% ≤62%	12%
Anti-Realist	91% ≥34%	88% ≥14%	100% ≥21%	75% ≥36%	72% ≥24%	99% ≥19%	86% ≥15%	98% ≥17%	91% ≥15%	87% ≥37%	88%

Most anti-realist responses were anti-realist in a cognitivist sense. On average, 73% of the responses to the abstract and 76% of the responses to the concrete truth-aptness tasks were in favor of moral sentences being truth-apt. Almost all other tasks showed an even higher proportion of cognitivism vis-à-vis non-cognitivism. In particular, participants’ responses dominantly indicated intuitions in favor of cultural relativism (36% in the abstract and 24% in the concrete condition) and individual subjectivism (25% in the abstract and 42% in the concrete conditions). Intuitions in favor of error theory, secular realism and theistic realism were less widespread (see Tables 3 and 4).

**Table 3:** Breakdown of realist and anti-realist responses in abstract measures (excluding truth-aptness task)

	Theory	Comparison	Metaphor	Disagreement	TOTAL
Secular Realism	20%	10%	13%	8%	13%
Theist Realism	12%	11%	11%	6%	10%
Cultural Relativism	27%	46%	53%	19%	36%
Individual Subjectivism	7%	28%	19%	47%	25%
Error Theory	4%	3%	4%	5%	4%
Non-Cognitivism	30%	3%		23%	19%

Our metaphor task did not discriminate between error theory and non-cognitivism (see Sec. 2.2.2). This is because we were not able to come up with a metaphor that clearly only reflected one but not the other of these views

**Table 4:** Breakdown of realist and anti-realist responses in concrete measures (excluding truth-aptness tasks)

	Martin Luther King	Wealth Distribution	Cheating	Overpricing Drugs	Physical Punishment	Immigration	Abortion	Racial Discrimination	Death Penalty	Golden Rule	TOTAL
Secular Realism	4%	10%	0%	17%	21%	0%	10%	0%	6%	7%	8%
Theist Realism	5%	1%	0%	10%	10%	1%	5%	3%	3%	6%	4%
Cultural Rel.	15%	21%	43%	19%	27%	18%	24%	41%	14%	23%	24%
Individual Subj.	35%	40%	34%	43%	33%	48%	59%	35%	51%	41%	42%
Error Theory	10%	10%	11%	6%	6%	15%	3%	8%	11%	10%	9%
Non-Cognitivism	35%	28%	12%	22%	24%	18%	9%	14%	21%	20%	20%

Even though subjects dominantly responded as anti-realists across all of our concrete disagreement tasks, there was nonetheless a significant spread between those tasks that received the least amount of anti-realist responses and those tasks that received the most, *t*(116) = 5.5, *p* < .001.

Also, similar to much previous research (e.g., Beebe and Sackris 2016; Goodwin and Darley 2008, 2012), participants’ strength of belief (i.e., the strength with which they agreed or disagreed with moral statementss) and their perception of consensus were also significantly higher for those concrete moral issues they had classified as realist than those they had classified as anti-realist, *t*s(46) = 4.7 and 5.9, *p*s < .001.

We did not find a significant correlation between secular realism and theistic realism (*r* = −.067, *p* = .47). This suggests that these views are not only conceptually distinct but also represent distinct psychological constructs.^20^

#### Inter- and Intrapersonal Variation

**Fig. 1:**
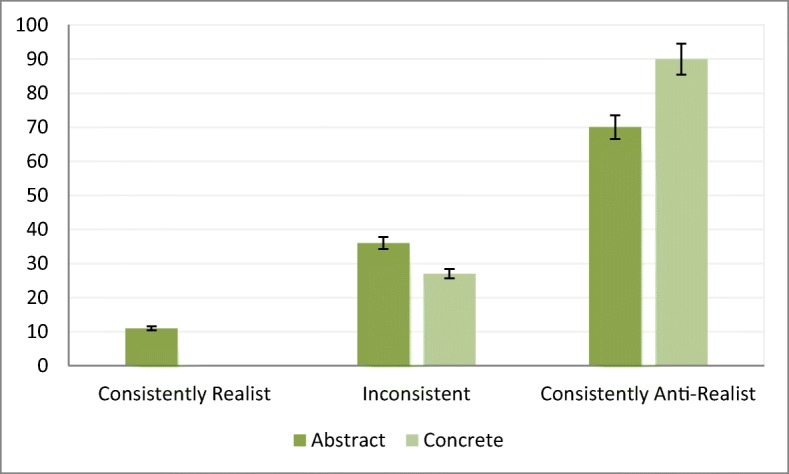
Number of participants who gave consistently realist, inconsistent and consistently anti-realist responses to the abstract and concrete measures

Above we defined a “consistent” participant as one who gives the same kind of responses in 100% of our abstract and at least 90% of our concrete tasks. None of our participants turned out consistently realist and 59 participants (50%) gave consistently anti-realist responses. This percentage is higher than what we would expect if people were answering randomly (binomial test, *p* = .016). Of the 58 participants who did not give consistent responses across both kinds of measures, 31 gave consistent anti-realist responses to the concrete measures (but not the abstract), 11 gave consistent anti-realist responses to the abstract measures (but not the concrete), and only 7 did not give consistent anti-realist responses across any of the two kinds of measures (Fig. 1).

Breaking participants’ responses down further, there were 18 people who gave consistent responses for all four abstract cases (4 for secular realism, 3 for theistic realism, 7 for cultural relativism, and 4 for individual subjectivism). Moreover, there were 14 people who gave consistent responses (at least 90%) for all ten concrete cases (3 for cultural relativism, 6 for individual subjectivism, 1 for error theory, and 4 for non-cognitivism).

When we grouped people’s responses to the concrete cases into individual subjectivism/cultural relativism and error theory/non-cognitivism, much more consistency emerged: 35 people (30%) gave consistent individual subjectivism/cultural relativism responses and 41 people (35%) gave consistent error theory/non-cognitivism responses. That is, many participants seemed to have the intuition that either moral truth is mainly determined by their own or by culturally dominant moral beliefs, or that there is no moral truth at all (see Tables 3 and 4).

There was a strong positive correlation between participants’ abstract/concrete realist responses and their abstract/concrete anti-realist responses, *r*s(117) = .52, *p* < .001 as well as a strong negative correlation between their concrete realist/abstract anti-realist and concrete anti-realist/abstract realist responses, *r*s(117) = −.52, *p* < .001. This suggests some consistency between abstract and concrete responses. Nonetheless, we did find a significant difference across the abstract and concrete measures. In the abstract tasks the anti-realist response rate was significantly lower than in the concrete ones (77% vs. 89%, *t*(116) = 4.4, *p* < .001). However, this abstract/concrete variation may not be genuine. It may actually simply be explained by differing responses to the disagreement tasks on the one hand and the theory, comparison and metaphor tasks on the other hand (see Tables 1 and 2).

#### Demographics

The strength of participants’ religious beliefs was negatively correlated with their anti-realist responses in both abstract and concrete conditions, *r*s(117) = −.34 and .30, *p*s < .001, as well as positively correlated with their realist responses, *r*s(117) = .34 and .30, *p*s < .001. As expected, this is driven almost entirely by participants’ *theist* realist responses. When they are removed, the correlations between realism and strength of religious beliefs drop to non-significance for the abstract cases and much lower significance for the concrete cases (*r*s(117) = .18 realist and − .18 anti-realist cases, *p*s = .048 and .050). These correlations suggest that realist/anti-realist intuitions are at least somewhat dependent on religiosity — a finding that is also supported by participants’ verbal explanations (and see also Goodwin and Darley 2008: 1356–1357; Yilmaz and Bahçekapili 2015).

Political orientation was correlated with participants’ abstract, but not concrete, responses — positively with their realist *r*(117) = .22, *p* = .017, and negatively with their anti-realist responses, *r*(117) = −.22, *p* = .017. This means the more conservative the person was the more realist their abstract intuitions were. Once again, these relationships went away when theist realism was removed, suggesting that the more conservative participants were more inclined towards theist realism than our more liberal participants.

## Discussion

As mentioned in the introduction to this article, previous research has converged on the hypothesis that ordinary people’s metaethical intuitions are pluralist in the sense that they regard moral realism as true with regard to some moral sentences or circumstances and anti-realism with regard to other moral sentences or circumstances (e.g., Beebe 2014; Beebe and Sackris 2016; Sarkissian et al. 2011; Wright et al. 2013, 2014). Our results cast some doubt on this hypothesis. Most of our participants’ intuitions did not vary across the realism/anti-realism distinction. They were rather consistently or overwhelmingly anti-realist. If at all, pluralism is only supported in a very anti-realism-leaning version.

Proponents of traditional metaethical pluralism may respond that there are plausible explanations of our findings that are consistent with their view. First, our low proportion of realist responses at the concrete level may be partially a function of the particular item statements that we used in our study (especially the lack of more “extreme” statements such as about murder or rape).^21^ Second, by informing participants upfront about the survey’s complexity and its attention and comprehension checks, as well as by excluding those who did badly on these checks, we may have biased our sample.^22^ And third, our efforts to make participants engage in reflection (by presenting them with explanations and requiring them to complete comprehension checks) may have had an effect too.

All of these explanations strike us as initially plausible. Yet, there are good reasons to believe that the best account of our divergent results will also appeal to our methodological improvements. Most importantly, we observed less realist responses and hence less traditional pluralism because we accounted for cultural relativism (W2), and did not wrongly identify cognitivism with realism (W3).

First, many previous studies have exclusively used intra-cultural disagreement scenarios. Participants’ “One person is right and the other one is wrong” responses to these scenarios were interpreted as being indicative of realism (e.g., Beebe 2014; Beebe and Sackris 2016; Goodwin and Darley 2008; Wainryb et al. 2004; Wright et al. 2013, 2014).^23^ But as long as the parties of a moral disagreement are members of one and the same culture not only realists, but cultural relativists should be drawn to this response as well (because in their view moral sentences are made true by the dominant moral beliefs within cultures; and within a particular culture there can only be one dominant moral belief about a moral sentence). Previous disagreement tasks hence likely exaggerated the proportion of realist responses.^24^

A second plausible explanation of our lower proportion of realist responses concerns our study’s truth-aptness tasks. So far “truth-apt” responses have exclusively been interpreted as being indicative of moral realism (e.g., Goodwin and Darley 2008; Wright et al. 2013, 2014). But again, this interpretation is inadequate. Cognitivist variants of anti-realism entail the truth-aptness of moral sentences as well. Subjectivists believe that moral sentences are true or false depending on whether they correctly represent the subjective moral facts (such as facts about individuals’ or culturally dominant moral beliefs); and error theorists believe that all moral sentences are false. Our study accounted for this fact by classifying “truth-apt” responses as reflecting *either* realism *or* cognitivist anti-realism. Thus, it raised the bar for providing a realist response in this regard as well.

To test these potential explanations, we returned to our data and did the following: First, we reinterpreted “One person is right and the other person is wrong” responses to our intra-cultural disagreement task as realist (regardless of how participants responded to the disagreement tasks that followed). In the abstract measures, this significantly shifted participants’ responses towards realism, from 13% to 35%, *t*(116) = 5.1, *p* < .001. In the concrete measures, it shifted them even more significantly towards realism, from 11% to 40%, *t*(116) = 10.8, *p* < .001. A similar finding emerged when we reinterpreted all truth-aptness responses as realist (if attributing truth-aptness) or anti-realist (if denying truth-aptness). In the abstract measure, this significantly shifted participants’ responses towards realism, from 23% realist to 33% realist, *t*(116) = 10.4, *p* < .001. In the concrete measure, the proportion of realist responses rose from only 11% to 44%, *t*(116) = 19.8, *p* < .001.

In our view, these considerations suggest that traditional metaethical pluralism might need to be replaced by what we will henceforth refer to as “anti-realist pluralism”. At least on a more reflective level most people’s metaethical intuitions may not vary much across the realism/anti-realism distinction. For most or even all moral issues they deny moral objectivity. At the same time our results also suggest, however, that there is significant intrapersonal variation *within* anti-realism. Specifically, many people seem to view the truth of moral sentences as depending on their own moral beliefs (individual subjectivism) and on the dominant moral beliefs within their culture (cultural relativism) (see Tables 3 and 4). Here are some representative explanations by our participants:

- The underlying theme between all of these answers [the participant’s answers] is that there is not one universal code of morality. I think that senses of morality come from several sources such as culture/society, or individual convictions shaped by personal experience.
- I think morality is dependent on a person’s personal opinion and the culture they grew up in.
- Along with the idea of culture as a construct for what a person’s morals may be with regard to certain topics it comes down to the individual and what they believe inside themselves is moral and immoral.

This within-anti-realism pluralism might be understood in two distinct ways. First, ordinary people may have the intuition that some moral sentences are made true by their own moral beliefs, while other moral sentences are made true by the dominant moral beliefs within their culture (“either-or pluralist anti-realist”). Second, it may seem to them that the truth of every moral sentence is determined by both their own moral beliefs and the dominant moral beliefs within their culture, but to varying degrees (“conciliatory pluralist anti-realism”). The prevalence of these distinct intuitions is to be settled by future research that employs a more fine-grained methodology.

Our argument for anti-realist pluralism is limited in two ways that we have already touched upon. First, some of our participants may not have assumed a correspondence-theoretic understanding of moral truth. It would be inappropriate to attribute intuitions in favor of anti-realist pluralism (or any other realist or anti-realist view) to these participants (Sec. 2.4). Second, our results also may not be widely generalizable. Not only may they be contingent on our item statements, our participants scoring high in traits such as need for cognition or general intelligence, and the presence of prior reflection; people from non-Western cultures may think differently about metaethics as well (see Henrich et al. 2010).

That said, we have gone some way in ruling out what has been probably the most serious objection leveled against research on folk moral realism, namely that participants systematically conflate the issue of moral objectivity with independent questions or may not have any determinate intuitions about it at all. These explanations strike us as implausible. We observed a higher proportion of anti-realist responses than we would expect if participants were answering randomly (Sec. 3.3.3). Some potentially distorting mental processes that might plausibly drive such a pattern can be (partly) ruled out. For example, participants’ responses likely are not attributable to emotional reactions (our design involved a number of abstract measures which half of the participants received first) or to their strength of agreement or perceptions of consensus (as they even responded in dominantly anti-realist ways to those moral sentences that they most strongly agreed to and took to be most widely accepted)*.*

There are also positive reasons for believing that our survey responses reflect determinate intuitions about moral objectivity. First, all analyzed participants paid at least minimal attention throughout the whole survey (as evinced by the fact that they passed our attention checks). Second, most of them showed a good understanding of the survey’s basic subject matter (as evinced by the fact that they performed well in our comprehension checks, see Sec. 3.3.1). Third, participants’ verbal explanations mostly at least roughly corresponded to their responses (at least in the sense that they reflected the responses’ basic realist or anti-realist direction). And fourth, having intuitions in favor of an individual subjectivism/cultural relativism brand of anti-realist pluralism makes sense from a psychological perspective. This view reflects and helps to navigate a central struggle in any person’s life: the struggle between individuality (a desire to be different) and conformity (a desire to fit in).

## Conclusion

In this paper we addressed the question of what ordinary people think about moral objectivity. Previous psychological research on this question has been subject to a number of methodological worries. Our aim thus was to advance this research, taking these worries into account. First, we proposed a new experimental design for measuring folk intuitions about moral objectivity. Then we reported and discussed the results of a survey that was based on this design. We found that most of our participants denied the existence of objective truths about most or all moral issues. In particular, many of them had the intuition that whether moral sentences are true depends both on their own moral beliefs and on the dominant moral beliefs within their culture (“anti-realist pluralism”).

What does this result imply for metaethics? We can think of several potential implications (such as for the conceptual question of what, if anything, moral sentences purport to refer to; see Pölzler and Wright under review). Here, however, we again focus on the presumptive argument that we introduced at the beginning of this paper.

Many philosophers accept that as ordinary people experience morality as a realm of objective truths, we have a prima facie reason to believe that it actually is such a realm (e.g., Dancy 1986; Enoch 2017a, 2017b; Huemer 2005; McNaughton 1988). This argument can be understood in various ways (see Loeb 2017b; Pölzler forthcoming). At least on the face of it, our results cast doubt on many of its versions. It just does not seem true that ordinary people (at least in the US) experience morality as a realm of objective truths — especially if they have given the issue some thought. In fact, if there were a philosophically valid way of inferring a presumption from metaethical intuitions then it would rather be appropriate to grant such a presumption to anti-realism.^25^

To this challenge many proponents of the presumptive argument will want to reply that is based on data on an irrelevant kind of intuitions. The intuitions that we measured appear to be both (1) reflective and tutored (because of our study’s instructions and comprehension exercises) and (2) explicit, i.e., such that participants were consciously aware of and minimally able to articulate them (because of the nature of our tasks and our verbal explanation requirement). In contrast, realists might reply, the presumptive argument only requires that people’s *pre-reflective*, *untutored* and *implicit* intuitions favor moral realism. Brink, for example, explains: “My appeal to commonsense moral thinking is not a prediction about the likely results of a Gallup poll on the issue of moral realism. Rather, my concern is with the philosophical implications or presuppositions of moral thought and practice” (1989: 25; see also, e.g., Enoch 2017a, 2017b).

This is a plausible reply that future research on folk moral realism should address heads-on (for first attempts see Pölzler et al. in preparation; Zijlstra under review). Yet, we doubt that it fully succeeds in deflecting the above challenge. To begin with, different versions of the presumptive argument entail hypotheses about different kinds of intuitions (Pölzler forthcoming). For many of these arguments the relevant kinds of intuitions (including their level of reflectiveness, tutoredness and implicitness; and the very meaning of these attributes) have not yet been sufficiently specified. Brink (1989), for example, only briefly and vaguely explains what he means by “the philosophical implications or presuppositions of moral thought and practice”. At one point one may even interpret him as suggesting that he is interested in reflective and tutored intuitions after all (1989: 25; see also Pölzler et al. in preparation).

Another worry about the reply is that prompting minimal reflection and providing minimal tutoring may actually be indispensable to research on folk moral realism. Without being subjected to these measures ordinary people may not have determinate intuitions about moral objectivity at all. Since the issue is so abstract and esoteric, such intuitions may rather only develop in the process of reflecting and being tutored. Moreover, suppose proponents of the presumptive argument are right that people do have determinate pre-reflective and untutored intuitions about moral objectivity. At the very least it seems difficult to validly *measure* these intuitions without prompting some reflection and providing some tutoring (as people so easily misunderstand questions about moral objectivity as being about their first-order moral intuitions, perceptions of social consensus, etc.; see W5, W10, and Pölzler 2018a, 2018b).^26^

Also note that in our study we used a number of different measures of moral realism. We agree that none of these measures may cover highly implicit intuitions. However, some may have gone at least some way. Both the metaphor and comparison tasks, for example, ask for — potentially unconscious — associations of morality with non-moral matters. Most importantly, our disagreement task scenarios are about a phenomenon that is part of everyday life, and do not involve any metaethical terminology (such as “objective”, “moral facts”, etc.). Such terminology is only used in the additional explanations of the answer options. However, when we removed these additional explanations in further independent research (thereby making the disagreement task even more implicit) our results more or less stayed the same, with participants’ responses still dominantly expressing intuitions in favor of moral anti-realism (Wright and Pölzler under review).

Finally, suppose our study indeed failed to measure those kinds of intuitions that are relevant to the presumptive argument. Still it seems that the above reply would fall short from rescuing this argument. It is surely *possible* that on a pre-reflective, untutored and implicit level ordinary people would side with moral realism. But why believe so? Proponents of the presumptive arguments have not yet provided any evidence in favor of that claim except from a small number of contested thought experiments (Enoch 2017b). Moreover, first attempts to measure more pre-reflective, untutored and implicit intuitions about moral objectivity (see Pölzler et al. in preparation; Zijlstra under review) suggest that most ordinary people might be anti-realist pluralists on this level too.

To sum up, then, there is reason to believe that our findings indeed pose a serious challenge to realists’ presumptive argument. This conclusion is important. So far even most anti-realists have accepted their presumptive disadvantage (e.g., Blackburn 2006: 153; Brink 1989: 24, 36; Mackie 2011: 35). This has strongly influenced the debate’s dialectic. While anti-realists have proposed numerous arguments against the existence of objective moral truths, realists have primarily only attempted to refute these arguments (e.g., Huemer 2005; Shafer-Landau 2006). Our study suggests that the time may have come to rethink the rules of this game. Metaethicists may have to take realism and anti-realism to at least start on an equal footing. Those who advocate moral objectivity may owe us convincing positive arguments in its favour; and those who deny it may be in a better position than has long been acknowledged.

## Footnotes

1 Some contemporary non-cognitivists argue that moral sentences are truth-apt in a deflationary sense (e.g., Blackburn 2000: 79). We will come back to this issue in Sec. 1.2 and 2.4 below. Moreover, error theory has often been restricted to particular kinds of moral sentences (such as positive ones) (e.g., Joyce 2007; Pigden 2007: 451), and has sometimes been stated in terms of these sentences being “untrue” rather than false (Joyce 2001: 6–9, 2007). For a plausible defense of my above formulation see Loeb 2007a.

2 This argument has been put forward in several different versions. For example, it has been claimed that realism has a presumption in its favor because our realist intuitions are best explained by realism (e.g., Brink 1989; Enoch 2017b), because these intuitions are best justified or vindicated by realism (e.g., Brink 1989; Shafer-Landau 2003), or because we have a prima facie reason to believe the content of any of our intuitions (e.g., Dancy 1986; Huemer 2005; McNaughton 1988). In what follows we will not be concerned with the argument’s philosophical merits but exclusively focus on its empirical premise.

3 Wainryb et al. used the labels “objectivism” and “relativism” to refer to claims that affirm/deny objective moral truths.

4 Goodwin and Darley used the labels “objectivism” and “subjectivism” to refer to claims that affirm/deny objective moral truths.

5 Studies involving subjects from different cultures include Beebe et al. (2015) and Sarkissian et al. (2011). By investigated subjects between the ages of 12 and 88 Beebe and Sackris (2016) also attempted to shed light on the question of how intuitions about the existence of objective moral truths vary across the lifespan.

6 Some researchers have successfully increased the variety of their item statements in some respects. Goodwin and Darley (2012), for example, included several statements about positive moral actions (in addition to the more usual statements about moral transgressions).

7 In the research mentioned above, one of us has argued that in addition to the studies by Goodwin and Darley and Wainryb et al., one or more of the following worries also apply to Beebe and Sackris 2016; Nichols 2004; Nichols and Folds-Bennett 2003; and Wright 2018. Moreover, the worries apply to any research that has adopted (part of) these studies’ methodology (such as Beebe et al. 2015; Goodwin and Darley 2012; Wright et al. 2013, 2014).

8 With regard to most worries, we explain how we tried to prevent them in the main text. The only worries that will not be explicitly mentioned are W4 (Conflation with Universalism/Relativism Debate) and W6 (Elicitation of Irrelevant Epistemic Intuitions). These worries were addressed by not including certain elements into our experimental design that some previous studies did include. In particular, we did not draw on certain elements of the moral/conventional task (e.g., Nichols and Folds-Bennett 2003) and we did not use epistemic terms such as “surely” or “could be” in formulating our answer options.

9 Our example of normative sentences about morality were: “It is wrong to break promises,” “The US has a duty to reduce its greenhouse gas emissions,” “Hitler was morally depraved,” and “Parents should be willing to make sacrifices for their children.” Our example of metaethical sentences were: “No person has any moral knowledge,” “There are facts about what is morally right or wrong, good or bad, virtuous or vicious, and so on,” “Sentences about what is the case never entail sentences about what morally ought to be case,” “Judgments such as ‘“Hitler was morally depraved’ are neither true nor false.”

10 To our knowledge, there is only one exception: a study by Zijlstra (under review) that is currently under review.

11 It may be objected that the formulation of this task biased subjects in favor of thinking that moral facts exist. However, while this may be true to some extent, note that the task also involved an answer option according to which moral facts are “illusions”; and that even if we exclude the metaphor task from our analysis this does not change our overall results, as reported in Sec. 3.3.

12 Half of the participants received a vignette in which the two people disagree about whether a situation is “good (right, virtuous, etc.)” (rather than “bad (wrong, vicious, etc.)”).

13 In a pilot study we found that if answer options are exclusively stated in terms of persons being right or wrong then participants often ignore or misunderstand their implications for the moral realism/anti-realism debate. It is therefore advisable to explicitly note these implications (as we did in parentheses).

14 Participants who receive our concrete measures first are provided with this explanation prior to the concrete truth-aptness task.

15 In particular, this explanation reads as follows: “The main point of some sentences is to make moral evaluations (i.e., evaluations about a moral matter, evaluating something as being morally right or wrong, good or bad). Here are some sentences that one might think belong to this category: It is wrong to break promises. The US has a duty to reduce its greenhouse gas emissions. Hitler was morally depraved. Parents should be willing to make sacrifices for their children. The main point of other sentences is to make other sorts of (non-moral) evaluations (i.e., evaluations that don’t have anything to do with morality, evaluating something as being correct or incorrect). Here are some sentences that one might think belong to this category: You put your shoe on the wrong foot. That chocolate ice cream tastes good. It is illegal for you to park on campus without a permit. It is rude to talk with your mouth full.”

16 To elaborate, in the disagreement task, both naturalist and non-naturalist realism is reflected by the following answer option: „One of these two people is right and the other one is wrong.“Compare this to many previous studies’ failure to include non-cognitivism. As long as all answer options state that either one or both of the parties of a disagreement are right (as, e.g., in Wainryb et al. 2004) participants who are drawn towards non-cognitivism have nowhere to go. They are forced to select an answer option that does not reflect their intuition (*neither* person is right or wrong) at all. This is a far bigger threat to validity than the failure to disentangle two positions that are jointly covered by one answer option. Hence, as explained above, in this study we primarily attempted to cover those metaethical views that we would expect the folk to hold, judging from class-room experience, conversations, etc.; or that must not be left out for the sake of minimal validity. Future research will hopefully employ experimental designs that also cover views such as response-dependence theory, naturalism and non-naturalism.

17 Often divine command theory is defended in a “restricted” sense: as the claim that only deontic moral properties (such as rightness) are dependent on the commands of God (e.g., Quinn 2000). Our study focused on the above comprehensive version (e.g., Evans 2013) because it seems more likely to be held by ordinary people. Also, note that according to some versions of divine command theory (better termed “theological voluntarism”), what matters are acts of divine will other than commanding. In this study, however, we assume a narrow conception that claims that moral facts are specifically identical to facts about what God commands us to do.

18 Classifying divine command theory as a variant of realism is also suggested by an ambiguity in our experimental design. Suppose we define this view as a variant of anti-realism. In our tasks participants were asked, very roughly, whether moral facts are determined by God’s commands. But participants who affirm this question may not think that (a) God’s commands are the ultimate grounds of morality. They may rather think that (b) God commands us to perform certain actions because he has insight into their independent goodness or rightness. If participants’ responses reflected (b) then they would have to be classified as realists even if divine command theory was defined as a variant of anti-realism. Requiring independence from only non-divine observers, in contrast, renders this problem moot (as subjects who choose that moral facts are determined by God’s commands can then be classified as realists independently of whether they assume (a) or (b)).

19 In this independent study, the statement „The earth is flat“was rated as realist by 55% of subjects, the statement „Boston (Massachusetts) is farther north than Miami (Florida)“was rated as realist by 63% of subjects, and the statement „The chemical formula of water molecules is H2O“was rated as realist by 60% of subjects. These numbers are of course lower than we would have hoped. That said, based on different measures, previous studies on folk moral realism found high proportions of scientific anti-realists too. In Nichols 2004 studies, for example, 13%, 23%, 22%, 23%, and 18% of subjects responded as anti-realists about facts (even though Nichols’ measures likely considerably exaggerated the proportion of realists by invoking first-order intuitions, see Pölzler 2018a, 2018b). This suggests that a considerable proportion of the population may genuinely hold that scientific facts are non-objective.

20 Based on different measures of moral realism, this distinctness has recently also been observed by Zijlstra under review.

21 Though note that even the cruelty of violent physical punishment of children was considered to be a non-objective matter by 79% of our participants; and many previous studies did not involve (many) such extreme statements either.

22 Though note that previous studies only found a correlation between participants’ disjunctive reasoning ability and their realist intuitions. Traits like need for cognition and reflective capacity did not correlate with these intuitions (Goodwin and Darley 2010)

23 The particular formulation of this response varied across studies.

24 This is supported by the fact that two studies that did include cross-cultural disagreement scenarios (Nichols 2004; Sarkissian et al. 2011) reported disproportionately strong tendencies towards anti-realism as well. Moreover, note that the above-mentioned intra-cultural disagreement scenarios also do not account for the possibility of participants being divine command theorists.

25 For objections against such inferences see again Loeb 2017b; Pölzler forthcoming.

26 In addition, reflective intuitions also seem to be more philosophically relevant when it comes to drawing potential semantic conclusions from research on folk moral realism (see Hannon 2018; Kauppinen 2007; Pölzler forthcoming).
